# Views and Attitudes of Blood Donors toward Blood Donation during the COVID-19 Pandemic in Thrace Region, Greece

**DOI:** 10.3390/ijerph19094963

**Published:** 2022-04-19

**Authors:** Christina Gkirtsou, Theocharis Konstantinidis, Dimitrios Cassimos, Eleni I. Konstantinidou, Eftychia G. Kontekaki, Viki Rekari, Eugenia Bezirtzoglou, Georges Martinis, Pantelis Stergiannis, for Thrace Study Group on Blood Donors’ Attitude during the COVID-19 Pandemic

**Affiliations:** 1 Blood Transfusion Center, University General Hospital of Alexandroupolis Dragana Campus, 68100 Alexandroupolis, Greece; gxristine@gmail.com (C.G.); kontekaki_e@hotmail.com (E.G.K.); geomimar@gmail.com (G.M.); 2 School of Social Sciences, The Hellenic Open University, 26335 Patra, Greece; pantstergiannis@yahoo.gr; 3 Pediatric Department, Democritus University of Thrace, 68100 Alexandroupolis, Greece; dkasimos@med.duth.gr; 4 Blood Transfusion Department, General Hospital of Drama, 66100 Drama, Greece; helenkonst28@gmail.com; 5 Blood Transfusion Department, General Hospital of Xanthi, 67100 Xanthi, Greece; vickyrekari@gmail.com; 6 Laboratory of Hygiene and Environmental Protection, Department of Medicine, Democritus University of Thrace, 68100 Alexandroupolis, Greece; empezirt@med.duth.gr

**Keywords:** blood donors, COVID-19, pandemic, attitude, community, transfusion

## Abstract

The COVID-19 pandemic has been going on for the last two years and it has affected our society and, amongst other things, has had a negative impact on blood donation, which has led to a significant reduction in blood supplies worldwide. The imposed restrictions in terms of physical presence and transportation, and the fear of the unknown, have aggravated the situation. In Greece, after the first cases of COVID-19 were reported, the blood supplies at the blood transfusion units (BTUs) were dramatically reduced. Although the blood transfusions were lessened during the COVID-19 pandemic period, the blood stocks at all the BTUs of the country were also reduced.

## 1. Introduction

In December 2019, a novel coronavirus was identified as an etiological agent of pneumonia outbreaks that emerged in Wuhan, China [1]. This coronavirus was named “severe acute respiratory syndrome coronavirus 2” (SARS-CoV-2), and the acute infectious disease that is caused by SARS-CoV-2 was classified as a novel coronavirus disease (COVID-19). On 11 March 2020, the World Health Organization (WHO) characterized the global health emergency of COVID-19 as a pandemic. SARS-CoV-2 infection can appear with many clinical manifestations, which range from asymptomatic to severe cases, and even to death [2]. The main route of SARS-CoV-2 transmission is person-to-person spread through secretions, such as saliva and respiratory droplets, which are expelled when an infected person coughs, sneezes, talks, or sings [3]. SARS-CoV-2 infection can be asymptomatic or accidentally asymptomatic, and individuals who are donating blood can be presymptomatic. Fortunately, the risk of transmitting SARS-CoV-2 by transfusion from presymptomatic donors is low [4,5].

The COVID-19 pandemic has been going on for the last two years, and it has affected many aspects of our society, and, among other effects, it has had a negative impact on blood donation, which has led to a significant reduction in blood supplies worldwide [6]. Nevertheless, an increase in total donations has been reported [7]. The restrictions on physical transportation and the fear of the unknown enemy could explain these findings. In Greece, after the first cases of COVID-19 were reported, the blood supplies at blood transfusion units (BTUs) were dramatically reduced. While the blood transfusions decreased during the pandemic period, the blood stocks at all the BTUs in the country were also reduced.

### Possible Impact of COVID-19 Pandemic on Transfusion Medicine

Blood transfusion (BT) has a significant impact on the saving of human lives under critical conditions. As a routine medical practice, BT improves the quality of life of patients with various chronic diseases. The increasing need for whole blood and its components can be observed worldwide. The gaps in the blood availability and transfusion safety are increased in low- and middle-income countries [8]. The situation deteriorated during the SARS-CoV-2 pandemic, and dangerous shortages of blood banks were reported in many countries [9,10]. According to the WHO, blood donors are usually volunteers and replacement donors. In some countries, there are also paid donors [11].

As the balance between the blood supply and the demand is very fragile, blood banks around the world search for more efficient ways to recruit new blood donors. The most frequent motives for blood donation worldwide are altruism, social responsibility, charity, and replacement donation [12,13]. Understanding the current patterns of the return rates and the motivations of direct blood donors will be helpful to enhancing the recruitment of blood donors. Blood donation carries some risk with regard to the interpersonal transfusion of blood products and cells. The rate of adverse events increases in relation to obesity, the male sex, and first-time donation. Vavic et al. report that first-time donors (FTDs) were more frightened, showed anticipatory anxiety, and were not sure that they would donate again. Moreover, younger donors were less satisfied with the staff’s behavior after donation [14]. Although we might expect that older persons would develop more frequent complications from the transfusion procedure, Goldman et al. report the opposite [15]. Evidence shows that, among routine blood donors, adverse events are either lower or the same in older donors compared with other donors.

The WHO has developed guidance on maintaining a safe and adequate blood supply during the COVID-19 pandemic [16]. According to this guidance, healthcare systems must do the following: (1) Mitigate the potential risk of COVID-19 transmission through the donor exposure to SARS-CoV-2; (2) Manage the demand for blood; (3) Ensure an uninterrupted supply of critical materials and equipment; and (4) Adequately inform donors, recipients, staff, and stockholders about the risk of infection transmission and the need to observe sanitary protocols, such as social distancing and wearing a mask [16,17].

Furthermore, as countries worldwide try to reduce the transmission rate of COVID-19 during blood donation by following the WHO guidelines, the psychosocial aspect of the pandemic that has affected blood donors has been evaluated as a significant factor [18,19].

In Greece, a national system for the recording of blood donors was established in 2014 and has been in full function ever since. This system also provides information regarding the transmissible infections of the donors, which empower us to avoid the transmission of transfusion-transmitted infections (TTI) to recipients. According to the national guidelines, all donors with active SARS-CoV-2 infection, or who are recovering from the disease, are deferred for at least 28 days after the resolution of the clinical signs and symptoms of the disease. Moreover, for donors who report possible contact with a suspected or confirmed COVID-19 case, as well as for donors who have travelled to countries with high epidemiological burdens of COVID-19, blood donation was postponed for at least 28 days after the exposure [20].

The aim of this study was to investigate the attitudes of donors in the region of Eastern Macedonia and Thrace, Northern Greece, during the COVID-19 pandemic. The second goal was to evaluate the demographic data of the blood donors in our region and to clarify their level of knowledge about blood donation and their motivations to donate blood. Furthermore, we recorded suggestions from blood donors for improving the BTU practice.

## 2. Materials and Methods

### 2.1. Study Design and Population

This was a multicenter questionnaire-based study that was carried out in Eastern Macedonia in the Thrace region of Northern Greece. The study questionnaire was distributed to all the BTUs in the region between January and March 2021. The demographic data of the study population are shown in Table 1. The following hospital BTUs participated in this multicenter study: University General Hospital of Alexandroupolis (Hospital 1); General “Sispanogleio” Hospital of Komotini (Hospital 2); General Hospital of Didymoteicho (Hospital 3); General Hospital Xanthi (Hospital 4); General Hospital of Drama (Hospital 5); and General Hospital of Kavala (Hospital 6). The expected number of participants in this study was 500 donors. Analytically, 479 questionnaires were distributed, and 416 of them were answered (Table 2).

### 2.2. Questionnaire

The questionnaire (see Supplementary Materials) had the following five sections: (1) Demographic data; (2) General knowledge about blood transfusion and personal history of blood donation; (3) Behavior and lifestyle trends; (4) Opinions/suggestions of donors on how to enhance donor recruitment; and (5) The donors’ attitudes toward blood donation during the COVID-19 crisis. Demographic data included age, gender, marital status, educational level, profession, and place of residence. Finally, we studied the effect of COVID-19 on the fears, worries, and anxieties among the blood donors. The fear and anxiety that are related to the COVID-19 scale were used, as previously described by Ahorsu et al. [21]. This assessment rates items on a five-point Likert-type scale, with each question scored from 1 to 5.

### 2.3. Ethical Committee

The study was approved by the local ethics and deontology committee in accordance with the Declaration of Helsinki (number 456/07-01-2021), and all participants provided written informed consent.

### 2.4. Statistical Analysis

For the statistical analysis, SPSS version 26 (SPSS Inc., Chicago, IL, USA) was used. The views and attitudes of the blood donors with regard to blood donation and the COVID-19 fear scale were evaluated by using chi-square (×2) and Pearson tests. The form of the scale distributions was checked by using the Blom (QQ plot) method, while their reliability coefficients were calculated by using the Cronbach method. Finally, through hierarchical models of multiple linear regression, the possible dependence of the attitude-and-behavior scale on the characteristics of the participating blood donors, their information, the data on blood donation, their opinions and suggestions on blood donation, and the COVID-19 fear scale was tested. A *p*-value of <0.05 was considered significant.

## 3. Results

All questionnaires were anonymously completed by blood donors in a few minutes. A total of 416 donors with a mean age of 37.8 ± 11.2 years participated in the study; 109 (26.2%) participants were female. The majority of respondents (203, 48.8%) had university or postgraduate degrees. The demographic data of the study population are shown in Table 1. The overall participation rate was 86.8%, with a good response from almost all the hospitals (Table 2).

**Figure 1:**
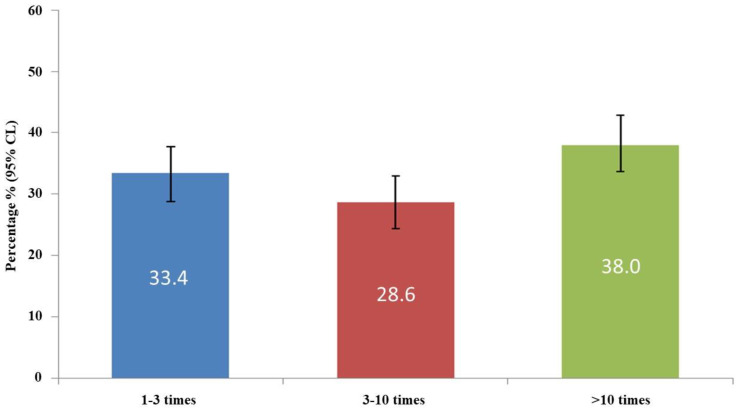
Frequency of blood donation.

With regard to the blood-donation history, 84.9% of the donors had donated blood in the past, and 38.0% of them had donated more than 10 times (Figure 1). Moreover, 64.4% were regular volunteers, while 55.3% had a volunteer blood-donor card. More than 50% of the participants had donors in their family, and only 12.0% had undergone a transfusion in the past. We also studied the frequency of blood donation in the preceding year and found that 142 (34.1%) had not donated blood for more than one year; the reasons are summarized in Supplementary Table S1. It is surprising that 39 donors (8.9%) intentionally hid some information before completing the procedure. The main reasons recorded were to ensure leave, to exclude sexually transmitted diseases, or to donate blood for replacement.

Statistically significant responses were recorded for the questions of Part C. A high percentage (94.5%) responded “agree or strongly agree” to the statement, “Donating blood can save human life”, and 85.4% to the statement, “As a donor, I feel confident and safe with the health services”. On the other hand, 70.2% responded “disagree or strongly disagree” to the statement, “I find that many issues or questions were personal or inappropriate”.

Among the reasons for discouraging blood donation, 81.4% of the respondents reported that they agreed or strongly agreed with a lack of empathy as a key factor. With regard to the lack of time as a factor, 43.5% of the study group disagreed or strongly disagreed, and 39.9% agreed or strongly agreed.

The factor that positively affects the intention to donate is the level of information about the need for blood: 95.7% agreed and 94.6% strongly agreed with informing citizens about the value of blood donation and about the need for blood.

With regard to the degree of concern or fear around COVID-19, there was a significant difference in the distribution of the responses to the seven statements. The vast majority of the respondents reported that they were not significantly influenced by the COVID-19 pandemic. In particular, they disagreed or strongly disagreed with the following statements: “Feeling afraid about COVID-19” (43.1%); “Makes me feel uncomfortable when I think about COVID-19” (49.3%); “I cannot sleep because I am worried about COVID-19” (88.4%); “When I watch news about COVID-19 on social media, I become nervous or anxious” (62.3%); “My heart beats loudly or my pulse races when I think that I will get COVID–19” (83.9%). The correlations of the attitude-and-behavior scales, the opinions and suggestions with regard to blood donation, and the scale of the fear due to COVID-19 are presented in (Supplementary Table S2). As shown in the table, the univariate correlations between the scales demonstrate significant agreement. The high score of the attitude-and-behavior scale is significantly related to the higher levels of the opinions and suggestions for improving the blood-donation process (r = 0.282, *p* < 0.05) and for enhancing blood donation (r = 0.383, *p* < 0.05). As expected for the opinion-and-suggestion scale, the four thematic units are significantly positively related to each other (*p* < 0.05), with a high score for improving the blood-donation process being associated with concerns, fears, and anxieties related to COVID-19 (r = 0.137, *p* < 0.05) (Supplementary Table S2).

A correlation of the scales of the attitudes and behaviors and the opinions and suggestions on blood donation, and the fear and anxiety related to COVID-19, in terms of the characteristics of the study participants, showed that the female sex was significantly associated with a higher degree of agreement with regard to the attitudes and behaviors around blood donation (r = 0.113, *p* < 0.05) and to the fear and anxiety related to COVID-19 (r = 0.114, *p* < 0.05). In addition, an older age was significantly associated with a higher degree of agreement with regard to the attitudes and behaviors around blood donation (r = 0.211, *p* < 0.05), and with a higher degree of disagreement with regard to distance dissuading people from donating blood (r = −0.099, *p* < 0.05). In addition, being employed in nonmanual work was significantly associated with higher agreement with distance as a dissuading factor (r = 0.159, *p* < 0.05), but also with fear and anxiety related to COVID-19 (r = 0.114, *p* < 0.05). A higher educational level was significantly associated with higher agreement with distance as a dissuading factor (r = 0.143, *p* < 0.05) (Supplementary Table S3).

In addition, according to the first regression model, it was found that a positive attitude toward blood donation was positively related to the female sex (β = 0.082, *p* = 0.031), an older age (β = 0.006, *p* < 0.001), and regular blood donation (β = 0.141, *p* < 0.001). These parameters were also positively related to the proposals for improving the blood-donation process (β = 0.117, *p* 0.001) and/or the recruitment of blood donors (β = 0.225, *p* < 0.001), and were negatively related to the views on the proposals for discouraging blood donation (β = −0.038, *p* = 0.046). In the second multivariate regression model, with the addition of the scale of the fear due to COVID-19 determined by the pandemic, it was found that the above parameters remained significantly positively related. The results do not seem to be significantly related to the fear and anxiety that is related to COVID-19 (β = −0.035, *p* > 0.05) (Table 3).

## 4. Discussion

The daily need for blood products is part of a dynamic equation, with many factors that are in relation to health services. Two main factors are blood usage and daily blood collection. The rapid spread of the COVID-19 pandemic created an urgent need for the reorganization of blood-transfusion services and the management of the blood supply. The implementation of lockdowns and social distancing led to a reduction in blood donations in our region. To avoid the dissemination of infection, donors with respiratory symptoms were discouraged from donating blood. In addition, potential donors were instructed to contact the blood-transfusion service if they developed any symptoms within the 14 days after donation. Thus far, in Greece, no transfusion-related transmission of COVID-19 has been reported. Even though SARS-CoV-2 seems to not have affected blood safety, the pandemic has negatively influenced the donation and utilization of blood components [22,23]. A retrospective study with regard to the utilization of blood components at 10 hospitals in Spain demonstrated that donations decreased 11% during the period of 1 to 15 March 2020 [22]. It was a pleasant surprise to come across a recent study by Gammon et al. that addresses an overall increase in blood donations. This result was attributed mainly to additional donor campaigns and to the measurement of antibodies to SARS-CoV-2 [7].

The present questionnaire-based research was conducted in order to examine the opinions and behaviors of blood donors with regard to blood donation and to improving the recruitment of new donors.

It was previously reported that the main motivation for blood donation was altruism [24,25,26]. This is line with the results of the present study. The volunteers were more often regular donors, and they donated more frequently per year compared to the replacement donors. Several studies note a significant relationship between the knowledge about and the attitude toward blood donation [27,28]. Likewise, in this study, increased agreement regarding the attitudes and behavior around blood donation was related to the level of knowledge. It was also significantly related to the female sex (β = 0.082, *p* = 0.031), an older age (β = 0.006, *p* < 0.001), to being a regular blood donor (β = 0.141, *p* < 0.001), and to a high degree of agreement with the proposals for improving the blood-donation process (β = 0.117, *p* < 0.001) Table 3.

It is well documented that epidemics have a great impact on human psychology. Nowadays, the media overwhelms us with huge amounts of information. The above, in conjunction with the socioeconomic background, have been the key factors for the psychological impact and mental health of the public during the COVID-19 pandemic. To prevent disinformation or rumors, and to reduce the negative impact on psychological health, the medical community and the governments need to provide responsibly accurate information regarding the COVID-19 pandemic [29] Wang et al., in a recent study that included 1210 respondents, report that half of the respondents rated the psychological impact of the COVID-19 pandemic as moderate-to-severe, and about one-third rated it as moderate-to-severe [30]. Bonichini and Tremolada conclude that, during the COVID-19 quarantine, there was the development of SARS-related post-traumatic stress symptoms (PTSD), which influence quality of life [31]. To prevent the negative impact of the COVID-19 pandemic, Wang et al. propose the use of smartphone-based psychoeducation and psychological interventions with cognitive behavioral therapy [30].

In this study, the blood donors that were examined had no concern with regard to COVID-19 transmission in relation to blood donation (Table 3). The above finding can be attributed to the preceding telephone communication of the medical staff with the possible blood donors. In the abovementioned communication, a thorough and in-depth analysis of the procedure of transfusion was performed, which clarified that COVID-19 does not spread by blood donation. In this study, the subjects who were enrolled were those who were convinced by the medical staff that blood donation is not risky for COVID-19 transmission.

A questionnaire-based study may have limitations. Some of the participants may not have provided true answers to some of the questions, which could subsequently affect the results and conclusions. Moreover, the study group was limited to donors who visited blood-transfusion services for donation, and not to the whole donor community. Furthermore, in this paper, there is a lack of questions about the knowledge and attitudes towards the donation of COVID-19 convalescent plasma as new therapeutic properties. In addition, the question about vaccination and COVID-19 was also disregarded.

## 5. Conclusions

The present study recorded an overall good level of knowledge about and positive attitudes toward blood donation among the donors in the Thrace area. The correlation of the scales of the attitudes and behavior and the opinions and suggestions on blood donation, and the fear due to COVID-19 in the study, showed that the higher scores on the opinions and suggestions for improving the blood-donation process are associated with increased fear and anxiety in relation to COVID-19. The measurement of anti-SARS-CoV-2 antibodies has proven to be very tempting for blood donors. It is crucial to implement reciprocal standards in order to maintain blood donation at satisfactory levels.

## Figures and Tables

**Table 1:** Demographic data.

Parameters	Participation	
	Number (N)	Percentage (%)
Age (Mean ± SD)	37.8 ± 11.2	
Gender		
Male	307	(73.8%)
Female	109	(26.2%)
Marital status		
Married	224	53.8
Single, divorced, or widowed	192	46.2
Profession		
Manual labor	92	22.1
Nonmanual labor	324	77.9
Education level		
Elementary school graduate	21	5.0
High school graduate	192	46.2
University degree	139	33.4
MSc or PhD degree	64	15.4
Residence		
Permanent resident	361	86.8
Temporary resident	54	13.0
Migrant	1	0.2
Place of residence		
Urban area (>2000 residents)	386	92.8
Rural area (<2000 residents)	30	7.2

**Table 2:** Participation rate. Hospital 1: University General Hospital of Alexandroupolis; Hospital 2: General “Sispanogleio” Hospital of Komotini; Hospital 3: General Hospital of Didymoteicho; Hospital 4: General Hospital Xanthi; Hospital 5: General Hospital of Drama; Hospital 6: General Hospital of Kavala.

	Total	Participants N (% of Total)	% of Participation
Hospital 1	299	299 (71.9)	100
Hospital 2	40	5 (1.2)	12.5
Hospital 3	40	13 (3,1)	43.3
Hospital 4	30	29 (7.0)	96.6
Hospital 5	40	40 (9.6)	100
Hospital 6	30	30 (7.2)	100
Total	479	416	86.8

**Table 3:** Hierarchical models of multiple linear regression of attitude-and-behavior scale in terms of characteristics of participating blood donors, their information and data on blood donation, opinions and suggestions, and fear and anxiety related to COVID-19.

	Attitude-and-Behavior Scale in Terms of Characteristics of Participating Blood Donors							
	Model 1				Model 2			
Factors	β	95% CL		*p*-Value	β	95% CL		*p*-Value
**Gender (female)**	0.082	0.008	0.157	0.031	0.081	0.006	0.156	0.035
**Age (years)**	0.006	0.003	0.009	<0.001	0.006	0.003	0.009	<0.001
**Educational level**	0.011	−0.030	0.053	0.588	0.011	−0.030	0.052	0.599
**Regular donor (1–2 times/year)**	0.141	0.071	0.210	<0.001	0.140	0.070	0.210	<0.001
**Opinions and suggestions on improving blood-donation process (higher score γ means greater agreement on opinions and suggestions)**	0.117	0.054	0.180	<0.001	0.116	0.053	0.179	<0.001
**Views and suggestions on distance preventing people from donating blood**	−0.021	−0.086	0.044	0.524	−0.020	−0.085	0.045	0.538
**Opinions and suggestions on attracting blood donors**	0.225	0.165	0.285	<0.001	0.224	0.164	0.285	<0.001
**Views and suggestions on being discouraged from donating blood**	−0.038	−0.075	−0.001	0.046	−0.038	−0.075	−0.001	0.044
**Fear** **and anxiety related** **to COVID-19 (highest score reflects highest agreement with fear and anxiety)**	-				0.008	−0.035	0.051	0.713
**R^2^ (adjusted R^2^)**	0.27 (0.26)				0.27 (0.25)			

## Data Availability

Not applicable.

## Supplementary Materials

The following supporting information can be downloaded at: https://www.mdpi.com/article/10.3390/ijerph19094963/s1, Table S1: Reasons for delaying blood donation; Table S2: Correlation of scales of attitude and behavior, opinions and suggestions on blood donation, and fear and anxiety related to COVID-19 of study participants (* *p* < 0.05); Table S3: Correlation of scales of attitudes and behaviors, opinions and suggestions on blood donation, and fear and anxiety related to COVID-19 in terms of characteristics of study participants (* *p* < 0.05); Questionnaire: Study of views and attitudes of blood donors toward blood donation during the COVID-19 pandemic.
