# Supplementary material for: Views and Attitudes of Blood Donors toward Blood Donation during the COVID-19 Pandemic in Thrace Region, Greece
Source: Int J Environ Res Public Health. 2022 Apr 19;19(9):4963. doi: 10.3390/ijerph19094963 (PMC9101185; doi:10.3390/ijerph19094963)
Supplement: Supplementary file 1 [file ijerph-19-04963-s001.zip › ijerph-1571931-supplementary.pdf]

Supplementary Materials

Title: Views and Attitudes of Blood Donors toward Blood Donation during the COVID-19 Pandemic in Thrace Region, Greece

Christina Gkirtsou [1,2], Theocharis Konstantinidis [1,*], Dimitrios Cassimos [3], Eleni I. Konstantinidou [4], Eftychia G. Kontekaki [1], Viki Rekari [5], Eugenia Bezirtzoglou [6], Georges Martinis [1], Pantelis Stergiannis [2] and for Thrace Study Group on Blood Donors' Attitude during the COVID-19 Pandemic [†]

Supplementary Table S1. Reasons for delaying blood donation.

Parameters	Participation	
	Number (N)	Percentage (%)
I had health problems	17	12.0
I was rejected as a donor	9	6.3
Staff misconduct	3	2.1
No one in immediate environment needed it	23	16.2
I had no encouragement/ reminder	44	31.0
I do not have time	20	14.1
There are no appropriate incentives	5	3.5
The last time I donated blood I had a problem, e.g., dizziness	7	4.9
Other reasons	14	9.9

Supplementary Table S2. Correlation of scales of attitude and behavior, opinions and suggestions on blood donation, and fear and anxiety related to COVID-19 of study participants (*p < 0.05).

	1	2	3	4	5
			Pearson (r)		
1. Attitudes an behavior around blood donation (higher score means greater agreement with more positive attitudes and behaviors)	--				
2. Opinions and suggestions for improving blood donation (higher score means greater agreement with more positive attitudes and behaviors)	0.282*				
3. Opinions and suggestions on distance	0.064	0.263*			

preventing people from donating blood					
4. Opinions and suggestions on attracting blood donors	0.383*	0.350*	0.259*		
5. Opinions and suggestions on discouraging blood donation	-0.053	0.180*	0.309*	0.123*	
6. Fear and anxiety related to COVID-19 (higher score means greater agreement with more positive attitudes and behaviors)	0.093	0.137*	0.013	0.077	0.085

Supplementary Table S3. Correlation of scales of attitudes and behaviors, opinions and suggestions on blood donation, and fear and anxiety related to COVID-19 in terms of characteristics of study participants (* $p < 0.05$).

	Gender	Age	Profession	Education
	Pearson (r)			
1. Attitudes and behaviors around blood donation (higher score means greater agreement with more positive attitudes and behaviors)	0.113*	0.211*	0.055	0.045
2. Opinions and suggestions on improving blood donation (higher score means greater agreement with more positive attitudes and behaviors)	0.061	0.090	0.048	0.045
3. Opinions and suggestions on distance as a factor dissuading people from donating blood	0.089	-0.099*	0.159*	0.143*
4. Opinions and suggestions on attracting blood donors	0.062	-0.069	-0.013	-0.008
5. Opinions and suggestions on being discouraged from donating blood	0.034	-0.149*	0.075	0.081
6. Fear and anxiety related to COVID-19 (higher score means greater agreement with more positive attitudes and behaviors)	0.114*	0.085	0.119*	0.061

Questionnaire

Study of views and attitudes of blood donors toward blood donation during the COVID-19 pandemic

Part A: Demographics data

Please enter the following information by selecting the appropriate box or writing the answer.

A1. Gender

Male 1

Female 2

A2. Year of birth

A3. Marital status

Single 1

Married 2

Divorced 3

Widow 4

A4. Profession

Manual labor or blue-collar worker (farmers, craftsmen, household etc.) 1

Non manual labor or white-collar worker (students, unemployed, retired, etc.) 2

A5. Place of residence

Urban area (> 10.000 residents) 1

Suburban area (2.000 - 10.000 residents) 2

Rural area (< 2.000 residents) 3

A6. Educational level

Without elementary school graduate	1
Non fully elementary school graduate	2
Elementary school graduate	3
Secondary educational graduate	4
High school graduate	5
Holder of Institute of Vocational Training Degree	6
Holder of Technological Educational Institute Degree	7
Holder of University Degree	8
Holder of Master of Science	9
PhD or PhD student	10
Other:	

A7. Residence

Permanent resident	1
Temporary resident	2
Migrant	3

Part B. General information

Please answer the questions by selecting the appropriate box

Question	No	Yes	I don't know	I don't answer
**B1. Are you an active voluntary non-remunerated blood donor? (who donate at least once a year)**	1	2	3	4
**B2. Are you a first-time donor (FTD)?**	1	2	3	4
**B3. Are you a member of a local blood bank association?**	1	2	3	4
**B4. Do you have a blood donor card?**	1	2	3	4
**B5. The motivated for blood donation is needs of transfusion for your family member or friend?**	1	2	3	4
**B6. Is someone in your immediate family environment a regular donor?**	1	2	3	4
**B7. Do you know your blood type?**	1	2	3	4
**B8. Have you ever had a blood transfusion?**	1	2	3	4
**B9. Do you donate blood for the first time for your family member or friends?**	1	2	3	4
**B10. Do you donate blood for the first time when you were soldier?**	1	2	3	4
**B11. Do you donate blood for the first time as a volunteer?**	1	2	3	4

If you have never donated blood proceed to Part C.

B12. The frequency of blood donation in the past.

1-3 times 1

3-10 times 2

More than 10 times 3

B13. I have not been donating blood for more than a year.

1. **No** 1

2. Yes *(Please answer the reason by selecting the appropriate box)* 2

(a) I have health problems 3

(b) I tried, but not accepted 4

(c) The bad behavior of the staff 5

(d) None from my family needed blood transfusion in the past 7

(e) No one in immediate environment (family or friends) needed transfusion 8

(f) I do not have free time (The hours or location of blood bank station is not comfortable) 9

(g) There are no appropriate motivations 10

(h) I had a problem during the last donated blood e.g. vertigo, fainting 11

(j) Lockdown due to COVID-19 12

(k) other _________________ 13

B14. I have hidden the truth in the questions before I give blood.

1. No 1

2. Yes (reason) 2

(a) Donated blood to receive a special reword from my work 3

(b) Donated blood for a friends or family member 4

(c) Donated blood to know my health status 5

(d) I did not consider my answer important for blood donation 7

(e) other: ______________________________ 8

Part C. Health Behavior and Lifestyle Trends among Donors

Please answer the questions by selecting the appropriate box.

Statements	**Absolutely disagree**	**Disagree**	**Midpoint (Neither agree nor disagree)**	**Agree**	**Strongly Agree**
**C1. Donating blood can have positive effects on my mental and physical health.**	1	2	3	4	5
**C2. Donating blood can save human life.**	1	2	3	4	5
**C3. Donating blood reserve, the day-off privilege.**	1	2	3	4	5
**C4. Understanding the motivation for encourage new blood donors.**	1	2	3	4	5
**C5. I became aware about blood donation when I once had to donate to a family member or friend.**	1	2	3	4	5
**C6. The need for blood in Greece has dramatically increased.**	1	2	3	4	5
**C7. The need for blood in Greece has increased during the Covid-19 pandemic (due to decreasing rate of blood donors).**	1	2	3	4	5
**C8. Blood supplies in Greece are insufficient due to the increasing of accidents.**	1	2	3	4	5
**C9. Blood supplies in Greece today are insufficient to cover the high transfusion needs.**	1	2	3	4	5
**C10. As a donor, I have a good level of knowledge about blood donation.**	1	2	3	4	5
**C11. I am informed about blood donation by the media (press, TF, social media).**	1	2	3	4	5
**C12. I am informed about blood donation through participation as volunteer.**	1	2	3	4	5
**C14. I am informed about blood donation through my personal involvement in gaining knowledge about blood donation.**	1	2	3	4	5

Statements	Absolutely disagree	Disagree	Midpoint (neither agree nor disagree)	Agree	Strongly Agree
**C15. Negative experiences (both personal or others) during blood donation are able to inhibit my blood donation behavior.**	1	2	3	4	5
**C16. Bud experiences due to staff error.**	1	2	3	4	5
**C17. The attitude of staff towards me during the las donation was particularly good and friendly.**	1	2	3	4	5
**C18. I am very satisfied with the infrastructure of the blood donation station**	1	2	3	4	5
**C19. The staff of blood donation station is very well trained.**	1	2	3	4	5
**C20. I find that many questions that are asked me before donating blood were personal or indistinguishable.**	1	2	3	4	5
**C21. In my opinion, the blood donation is important.**	1	2	3	4	5
**C22. I have often offer volunteer work and / or I belong to associations with voluntary activity**	1	2	3	4	5
**C23. If my health allows it, I want to remain a blood donor until the upper age limit in 65 yrs.**	1	2	3	4	5
**C24. As donor, I feel confident and safe with the health services.**	1	2	3	4	5
**C25. Blood donor counseling has a positive impact to avoiding high-risk behavior during the Covid-19 pandemic.**	1	2	3	4	5

Part D: Opinions - Suggestions

Please answer the questions by selecting the appropriate box.

**D1. The blood donation process can be improved by:**	**Absolutely disagree**	**Disagree**	**Midpoint (Neither agree nor disagree)**	**Agree**	**Strongly Agree**
Reducing the waiting time	1	2	3	4	5
Increasing mobile units and blood donation stations	1	2	3	4	5
Improving of promotion and information the blood donation	1	2	3	4	5
Improving the infrastructure of blood transfusion department.	1	2	3	4	5
Increasing the medical staff of blood transfusion department.	1	2	3	4	5
Improving of flexibility in schedules of blood transfusion department	1	2	3	4	5
Improving of organization and function of blood transfusion departments in Healthcare Units.	1	2	3	4	5

**D2. Why do you think a person has never been blood donation?** *(Please answer the questions by selecting the appropriate box that most closely matches on your opinion.)*	**Absolutely disagree**	**Disagree**	**Midpoint (Neither agree nor disagree)**	**Agree**	**Strongly Agree**
Lack of time	1	2	3	4	5
Social Indifference	1	2	3	4	5
Psychological phobias of blood donation	1	2	3	4	5
Fear of transmitting a disease	1	2	3	4	5
Health problem	1	2	3	4	5
Lack of information about where and how blood donation takes place	1	2	3	4	5
Lack of confidence in health care services affects blood donors' mobility and compliance during the Covid-19 pandemic	1	2	3	4	5
No reason	1	2	3	4	5

**D3. How do you think the blood donors' mobility and compliance could increase during the Covid-19 pandemic?** *(Please answer the questions by selecting the appropriate box that most closely matches on your opinion)*	**Absolutely disagree**	**Disagree**	**Midpoint (Neither agree nor disagree)**	**Agree**	**Strongly Agree**
By increasing information on social media.	1	2	3	4	5
By informing of the citizens why is blood donation is important in our society.	1	2	3	4	5
By Informing of the citizens about the needs of blood.	1	2	3	4	5
By policy measures taken against the spread of infection: providing Personal Protective Equipment (PPE) e.g., mask	1	2	3	4	5
By providing free and safe movement to the blood donation station.	1	2	3	4	5
By providing free rapid test for the Covid-19.	1	2	3	4	5
By providing free antibody test for the Covid-19.	1	2	3	4	5

**D4. Factors that hinder blood donation (negative motivations)** *(Please answer the questions by selecting the appropriate box that most closely matches on your opinion)*	**Absolutely disagree**	**Disagree**	**Midpoint (Neither agree nor disagree)**	**Agree**	**Strongly Agree**
Fear of needles.	1	2	3	4	5
Fear of seeing blood (Hemophobia).	1	2	3	4	5
Fear of pain (Algophobia).	1	2	3	4	5
Fear of faint before a donation.	1	2	3	4	5
The fear of contracting Hepatitis or AIDS during the donating blood.	1	2	3	4	5
Fear of getting blood test results.	1	2	3	4	5
Donating blood take so long.	1	2	3	4	5
Fears and Anxiety around the Covid-19.	1	2	3	4	5
Another cause for fear.	1	2	3	4	5

Thank you very much for your participation and cooperation!
